# Implicit but not explicit extinction to threat‐conditioned stimulus prevents spontaneous recovery of threat‐potentiated startle responses in humans

**DOI:** 10.1002/brb3.1157

**Published:** 2018-12-04

**Authors:** Javiera P. Oyarzún, Estela Càmara, Sid Kouider, Lluis Fuentemilla, Ruth de Diego‐Balaguer

**Affiliations:** ^1^ Department of Cognition, Development and Educational Psychology University of Barcelona Barcelona Spain; ^2^ Cognition and Brain Plasticity Group, IDIBELL Bellvitge Biomedical Research Institute Barcelona Spain; ^3^ Brain and Consciousness Group, Département d'Études Cognitives, École Normale Supérieure PSL Research University Paris France; ^4^ Institute of Neurosciences University of Barcelona Barcelona Spain; ^5^ Catalan Institution for Research and Advanced Studies ICREA Barcelona Spain; ^6^ Present address: Psychology Department New York University New York New York

**Keywords:** electrodermal activity, extinction learning, fear conditioning, implicit extinction, skin conductance response, threat conditioning, threat‐potentiated startle responses

## Abstract

**Introduction:**

It has long been posited that threat learning operates and forms under an affective and a cognitive learning system that is supported by different brain circuits. A primary drawback in exposure‐based therapies is the high rate of relapse that occurs when higher order areas fail to inhibit responses driven by the defensive circuit. It has been shown that implicit exposure of fearful stimuli leads to a long‐lasting reduction in avoidance behavior in patients with phobia. Despite the potential benefits of this approach in the treatment of phobias and posttraumatic stress disorder, implicit extinction is still underinvestigated.

**Methods:**

Two groups of healthy participants were threat conditioned. The following day, extinction training was conducted using a stereoscope. One group of participants was explicitly exposed with the threat‐conditioned image, while the other group was implicitly exposed using a continuous flash suppression (CFS) technique. On the third day, we tested the spontaneous recovery of defensive responses using explicit presentations of the images.

**Results:**

On the third day, we found that only the implicit extinction group showed reduced spontaneous recovery of defensive responses to the threat‐conditioned stimulus, measured by threat‐potentiated startle responses but not by the electrodermal activity.

**Conclusion:**

Our results suggest that implicit extinction using CFS might facilitate the modulation of the affective component of fearful memories, attenuating its expression after 24 hr. The limitations of the CFS technique using threatful stimuli urge the development of new strategies to improve implicit presentations and circumvent such limitations. Our study encourages further investigations of implicit extinction as a potential therapeutic target to further advance exposure‐based psychotherapies.

## 1 INTRODUCTION

The ability to learn that previously threatening stimuli are no longer a threat is critical for mental health since the disruption of this process can lead to anxiety disorders such as phobias and post‐traumatic stress disorder, PTSD. A long‐standing critical issue in the treatment of threat‐related memories is the high rate of relapse after initially successful therapy (Craske & Mystkowski, 2006).

It has been established that threat learning operates and forms supported by two distinct brain circuits (Hamm & Vaitl, 1996; LeDoux, 1993). The first is an affective learning system grounded in the defensive circuit based in the amygdala and operating implicitly (LeDoux, 1993). The second is a cognitive learning system, associated with the acquisition of the declarative knowledge of stimuli contingencies, expectancy of threat, and conscious experience of fear that is sustained by hippocampal and prefrontal brain areas (Baeyens, Eelen, & Crombez, 1995; Lang, Davis, & Öhman, 2000; LeDoux & Brown, 2017; Purkis & Lipp, 2001).

Exposure‐based therapy is the most used procedure to treat threat‐related memories (Rothbaum & Davis, 2003) and is founded on the principles of extinction learning (Craske, 1999; Milad & Quirk, 2012) where the threat‐predicting stimulus (i.e., conditioned stimulus, CS) is repeatedly presented in the absence of the negative outcome (e.g., unconditioned stimulus, US). Through this procedure, subjects learn an inhibitory memory that, relying on prefrontal structures (e.g., dorsolateral and ventromedial prefrontal cortex) (Phelps, Delgado, Nearing, & LeDoux, 2004; Schiller, Kanen, LeDoux, Monfils, & Phelps, 2013), suppresses the expression of the defensive responses initiated by amygdala‐subcortical structures (Pare & Duvarci, 2012; Sotres‐Bayon, Cain, & LeDoux, 2006). However, this inhibitory function often fails, and defensive responses are spontaneously recovered with the passage of time (Rescorla, 2004).

It has been suggested that since extinction learning leaves the affective memory fairly intact (Baeyens et al., 1995; Myers & Davis, 2002), such implicit trace could later motivate fear recovery, especially when the inhibitory structures (i.e., the prefrontal cortex) are impaired, as is the case with anxiety‐related patients (Konarski, Mcintyre, Soczynska, & Kennedy, 2007; Sotres‐Bayon et al., 2006), or under stressful situations (Jacobs & Nadel, 1985). Some studies have shown that procedures that avoid prefrontal cortex (PFC) engagement to inhibit threat‐related memories are highly effective in preventing the recovery of defensive responses to threat‐conditioned or phobic stimuli (Koizumi et al., 2016; Schiller et al., 2013; Siegel & Weinberger, 2009). Of particular interest are the works of Siegel and Weinberg (Siegel & Warren, 2013a, 2013b) showing that very brief repeated masked exposure to phobic stimuli led to a long‐lasting reduction in avoidance behavior in spider phobics. In a recent fMRI study, the authors (Siegel et al., 2017) suggested that the beneficial effects of masked exposure might have been mediated through a facilitation of threat memory processing and the activation of regulation areas as participants do not experience subjective distress during exposure. To date, however, implicit extinction is still underinvestigated, harboring important theoretical as well as clinical implications.

Here, we investigated the effects of implicit extinction on a fearful memory after 24 hr, using a continuous flash suppression technique (CFS). To model fear acquisition and exposure‐based therapy, healthy participants were threat conditioned on day 1 to fearful faces. On day 2, stimuli were presented through a stereoscope, either invisibly (through CFS) for the implicit group or explicitly for the explicit group. On day 3, participants were normally presented to threat‐conditioned stimuli and recovery of defensive responses was tested by analyzing threat‐potentiated startle responses, electrodermal activity, and online expectancy reports.

As it has been suggested by other authors (“Anxious: The Modern Mind in the Age of Anxiety by Joseph E LeDoux, book review”, 2015; Brewin, 2001; Siegel et al., 2017), we predict that by restraining cognitive‐mediated fear processing, implicit extinction would promote threat memory processing at the implicit level and hinder the recovery of defensive responses.

## 2 MATERIALS AND METHODS

### 2.1 Participants

#### 2.1.1 Implicit group

Fifty‐nine (46 women, *M = *22.95 years, *SD *= 3.78) healthy students with normal or corrected‐to‐normal vision were recruited for this group. On the first day, we excluded 16 participants that did not meet the threat acquisition criteria (see Inclusion criteria for acquisition). From these, 23 more participants were excluded on the second day because they broke the suppression effect during implicit extinction (see exclusion criteria for image suppression). A final sample of 20 participants fulfilled the criteria for inclusion and followed the three consecutive‐days experimental protocol of the implicit group.

#### 2.1.2 Explicit group

Thirty‐two healthy students with normal or corrected‐to‐normal vision were recruited for this group (25 women, *M = *20.5 years, *SD *= 2.39). On day 1, we excluded 13 participants that were not threat conditioned and three that were nonresponders (see Inclusion criteria for acquisition). One participant did not return for day 3. A final sample of 15 participants fulfilled the criteria for inclusion and followed the three consecutive‐days experimental protocol of the explicit group.

The study was approved by the Institute of Biomedical Research of Bellvitge ethics committee, and all subjects from both groups signed an informed consent before their participation.

### 2.2 Psychological inventories

In order to control for psychological individual differences that could influence threat learning, all participants completed the Spanish version of the Spielberger State‐Trait (STAI‐T), the State‐State (STAI‐S) Anxiety Inventory (Spielberger, 1983), and the Spanish version of the 25‐item English Resilience Scale (Wagnild & Young, 1993) containing the “Acceptance of Self and Life” (ASL) and “Personal Competence” (PC) subscales.

### 2.3 Stimuli

#### 2.3.1 Visual stimuli

We employed Ekman's fearful faces (Ekman, 1976) as the conditioning stimuli (CS) as they can be processed in the absence of awareness through a rapid subcortical amygdala route (McFadyen, Mermillod, Mattingley, Halász, & Garrido, 2017). Faces were presented for 5 s with intertrial intervals (ITI) of 10–12 s (after electrodermal activity was stabilized). Stimulus order presentation was randomized with the constraint that no more than three consecutive repetitions of the same stimuli occurred. Stimuli were displayed on a 22‐inch computer monitor (resolution = 1,024 × 768 pixels; refresh rate =60 Hz) and were controlled using Psychophysics Toolbox software (Brainard, 1997; Pelli, 1997). Stimulus contrast was equally set for all participants, at a level that was clearly visible when viewed on its own but was also easily suppressed with continuous flash suppression (CFS; see CFS in Experimental task below).

#### 2.3.2 Electrical stimulation

We used a mild electric shock to the wrist as the unconditioned stimulus (US) during threat conditioning on day 1. Shocks were delivered through an electrode attached with a Velcro strap to participants’ dominant inner wrist, with a maximum intensity of 15 mA and 50‐ms duration and coterminated with faces presentation (Oyarzún et al., 2012). A Grass Medical Instruments stimulator (Grass S48 Square Pulse Stimulator) charged by a stabilized current was used with a Photoelectric Stimulus Isolation Unit (Model PSIU6). At the beginning of the session, participants regulated shock intensity to a level which they described as very uncomfortable yet not painful.

#### 2.3.3 Airpuffs

In order to measure threat‐potentiated startle responses, we mechanically provoked blink responses by delivering 40‐ms airpuffs, through a hosepipe directed to the anterior part of the temporal region between the outer canthus of the eye and the anterior margin of the auditory meatus (Haerich, 1994; Hawk & Cook, 1997) of the dominant‐hand side. Airpuffs were delivered 4.5 s after every face presentation onset (did not overlap with electrical stimulations) and during every other intertrial interval (ITI). In order to habituate subjects to airpuff stimulation, each day started with 10 startle probes (Sevenster, Beckers, & Kindt, 2012a).

### 2.4 Experimental task

#### 2.4.1 Day 1. Fear acquisition

**Figure 1:**
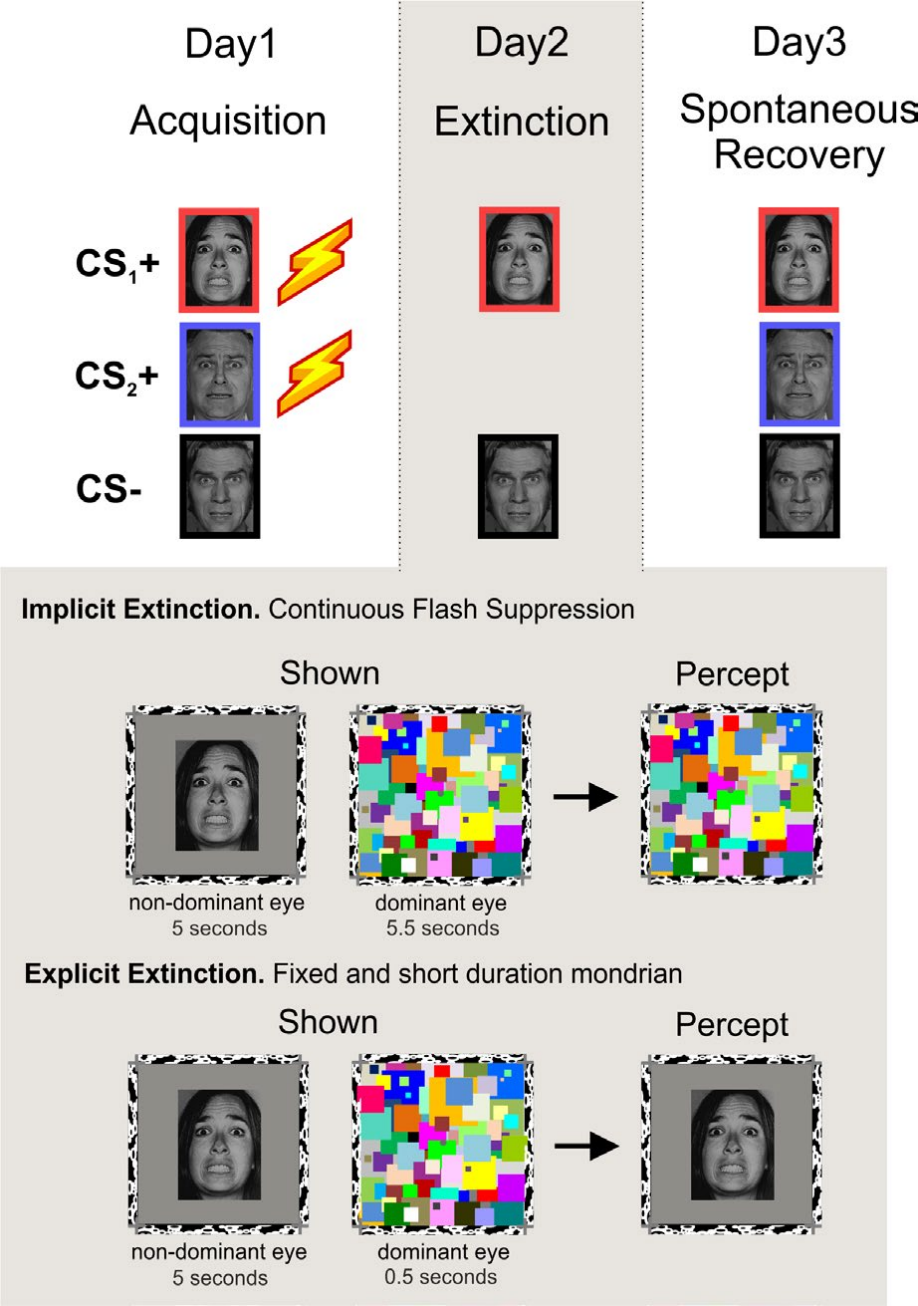
Three‐day experimental design: acquisition extinction and spontaneous recovery test. Two faces were fear‐conditioned on day 1 (CS_1_+ and CS_2_+), whereas a third face served as the neutral stimulus NS. CS_1_+ and the CS− were presented with no reinforcement on the second day using a continuous flash suppression (CFS) setting (with a stereoscope and colorful patches). These Mondrians were continuously flashing during picture presentation in the implicit group but were fixed and briefly presented in the explicit group. Acquisition on day 1 and recovery test on day 3 were conducted explicitly with faces at the center of the screen and without CFS setting

On day 1, participants were randomly presented with three fearful faces, eight times each. Two of them (CS_1_+ and CS_2_+) coterminated with a mild electric shock to the wrist on 75% of the trials (reinforcement was omitted in the 1st and 5th trial), and a third one was never followed by the aversive stimulus (neutral stimulus, CS−). Face gender was counterbalanced and randomized across participants. To acquire asymptotic levels of learning, participants were instructed that two faces were going to be followed, most of the time, by an electric shock, while the third one was safe (Figure 1).

##### 2.4.1.1 Inclusion criteria for acquisition

The first inclusion criterion aimed to ensure that participants were fear‐conditioned. We selected participants that showed differential electrodermal activity and startle potentiation to both threat‐conditioned stimuli compared to neutral; that is, the average of the final four trials, in the acquisition session, for both CSs+ was greater than for the CS− stimulus in the electrodermal activity (EDA) or startle responses (SR) index. In addition, we excluded non‐responder participants who showed below 0.02 µS peak‐to‐peak amplitude in the EDA index in more than 75% of unreinforced trials during acquisition (Raio, Carmel, Carrasco, & Phelps, 2012).

#### 2.4.2 Day 2. Extinction session

##### Implicit extinction group

Twenty‐four hours after threat conditioning, using a stereoscope and the CFS technique (see CFS below), participants were unconsciously exposed with only two of the images presented on day 1: CS_1_+ and CS−, 16 times each in the absence of electric shocks. In order to control for participants’ awareness of the face presentation, we asked for a subjective report using the keyboard arrows. After each trial, they were asked: “Do you think you might have seen a face?” “Yes” or “No,” and then “Was it a male or a female?” Subjects then indicated “male” or “female” and how sure they were of their answer with “sure” or “not sure.”

###### Detection task

In order to dissuade participants to voluntary explore the nondominant eye (by closing one eye) and thus break the CFS effect, we included a simple detection task on the dominant eye during Mondrian display (see CFS below). Three seconds after Mondrian onset, a central gray dot would randomly change to a different color for 1 s. At the end of the three awareness questions, participants had to answer whether the dot had turned to green or not; although no feedback was received after each response, participants were encouraged to be accurate in this task. Participants were pretrained for this task in the training session (see training session below).

###### Exclusion criterion for image suppression

To ensure full image suppression, we excluded participants that answered, in at least one trial: “yes” to the first question (“Do you think you might have seen a face?”) and were correct and confident (answered “sure”) in indicating the gender of the perceived face. Following this selection criteria, all the participants included in the final sample reported not seeing anything besides the Mondrian at all trials; that is, for every trial, the participants included in the sample answered “No” to the first question (except for one subject that answered seeing something on one trial), they all guessed faces at chance in the second question (main percentage of hits 46.71%; *SD *= 7.5; [34.38%–58.06%]) and responded to be “not sure” about the guess in the third question. Participants learned to answer these three awareness questions during the training session on day 2.

###### Continuous flash suppression

We employed the continuous flash suppression (CFS) technique, a binocular rivalry‐based method capable of reliably suppressing visual awareness despite stimulus presentation for long periods of time (Fang & He, 2005; Lin & He, 2009; Tsuchiya & Koch, 2005). Using a mirror stereoscope (Stereoaids, Australia) placed 45 cm from the screen, we presented a continuously flashing colorful pattern (Mondrian; at 10 Hz) to the dominant eye and low‐contrast (albeit visible) faces to the other, nondominant eye. Mondrians were created with MATLAB (MathWorks, Natick, MA, USA) and the Psychtoolbox (Brainard, 1997; Pelli, 1997) and were presented for 5.5 s, starting 500 ms before face onset. In this manner, target faces were rendered invisible to the participants and thus processed without awareness. To determine eye dominance, we used a sighting dominance test (Porac & Coren, 1976) where we asked participants to hold, with extended arms, a plastic board and look through a central small aperture to a picture placed on the wall at a 2‐m distance. The investigator would then cover one participants’ eye at a time and ask for a subjective report of the image. If the image was no longer seen when covering a certain eye, then that eye was considered dominant.

###### Training session

After the eye dominance test and before starting the experiment on day 2, participants had a training session for 5 min to calibrate the stereoscope, ensure image suppression and familiarize participants with the task and questions. First, a black and white image of a zebra was presented to one eye and the zebra outline was presented to the other. The subjects adjusted the mirrors of the stereoscope using two knobs so that each eye in isolation saw either the full zebra or the full zebra outline, and with two eyes, the zebra was aligned within the zebra outline. Then, subjects in both groups initiated a training sequence using six presentations of random objects (instead of the faces) where they were familiarized with the three awareness questions and, in the case of the implicit group, with the detection task.

##### Explicit extinction group

Participants followed the same procedure as the implicit group (same number, ITI, and length of stimuli presented through the stereoscope). However, for this group, face pictures were explicitly presented. Mondrians were presented (in the dominant eye) for only 500 ms before face‐picture presentation, so faces were fully visible to the participants, for the following 5 s (the same duration as in the implicit group), in the nondominant eye. In the same way, as with the implicit group, the same three questions regarding picture awareness followed each image presentation. In order to encourage participants to pay attention to faces presentation, this group did not perform the color detection task. All participants reported seeing the faces at all trials; that is, they answered “Yes” to the first question (except for one subject that reported not seeing a face on one trial), presented 100% accuracy in gender detection and were always sure about their response.

#### 2.4.3 Day 3

##### Spontaneous recovery test

After 24 hr, we tested for recovery of defensive responses to all stimuli. Participants were presented with the three faces they saw on the first day, six times each in the absence of the shock. To remove attentional orienting effects on the first trials, an extra presentation of the neutral stimulus, which was not included in the analysis, was presented at the beginning of this session.

##### Online threat expectancy ratings

During the spontaneous recovery test, participants had to indicate whether they expected to receive, or not, an electric shock after seeing each face on the screen. One second after face presentation, the question “Are you expecting to receive a shock?” appeared on the screen for 3 s. Participants answered, using the arrows of the keyboard, “Yes,” “No” or “I don't know.”

### 2.5 Measures

#### 2.5.1 Threat‐potentiated startle responses

Startle responses were analyzed after delivery of airpuffs. We performed a monocular electromyography (EMG) on the orbicularis ocular muscle of the dominant eye. A 6 mm Ag/AgCl electrode filled with a conductive gel was placed 1.5 cm below the lower eyelid in line with the pupil at forward gaze, a second electrode was placed 2 cm lateral to the first one (center‐to‐center), and a signal ground electrode was placed on the forehead 2 cm below the hairline (Blumenthal et al., 2005).

##### EMG data analysis for SR

Raw EMG data were notched and band‐pass filtered (28–500 Hz, Butterworth, 4th order), and afterward rectified (converting data points into absolute values) and smoothed (low‐pass filter 40 Hz) (Blumenthal et al., 2005). Peak blink amplitude was determined in a 30‐ to 150‐ms interval following airpuff delivery. EMG values were standardized using within‐participant *Z* scores for each day, and outliers (*Z* > 3) were replaced by a linear trend at point (Sevenster et al., 2012a). For comparisons between extinction on day 2 and spontaneous recovery test on day 3, *Z* scores were calculated using both extinction and recovery test data. For comparisons within stimuli (CS_1_+, CS_2_+, and CS−) on day 3, *Z* scores were calculated using only recovery test data.

#### 2.5.2 Electrodermal activity

Electrodermal activity and EMG were sampled at 1,000 Hz and were recorded during the whole session using BrainAmp amplifiers (Brain Products). EDA was assessed using two Ag‐AgCl electrodes attached to the middle and index fingers of the nondominant hand.

##### EDA data analysis

Electrodermal activity waveforms were low‐pass filtered (1 Hz) and analyzed offline with MATLAB 7.7. F. Single‐trial changes in EDA were determined by taking the base‐to‐peak difference for a 4.5 s window after stimulus onset and before airpuff (or electric shock) delivery. The resulting amplitude of the skin conductance response (SCR) value was standardized using within‐participant *Z* scores for each day, and outliers (*Z* > 3) were replaced by a linear trend at point (Sevenster et al., 2012a). As for EMG analyses, comparisons between extinction on day 2 and spontaneous recovery test on day 3 used *Z* scores calculated using both extinction and recovery test data. For comparisons within stimuli (CS_1_+, CS_2_+, and CS−) on day 3, *Z* scores were calculated using only recovery test data.

#### 2.5.3 Online US‐expectancy ratings (OER)

Since explicit evaluation of contingencies could affect learning during fear acquisition and extinction learning, expectancy ratings were made only during day 3. After each image presentation, the question “Are you expecting to receive an electrical shock?” appeared on the top of the screen for 3.5 s to which participants answered “Yes” (scored 3), “No” (scored 1) or “I don't know” (scored 2) using the keyboard. Participants were encouraged to maintain their hands over the keyboard at all times and to restrict hand and head movement as much as possible.

## 3 RESULTS

### 3.1 Acquisition

Equivalent levels of threat acquisition for conditioned stimuli in both groups and in both measures.

#### 3.1.1 Threat‐potentiated startle responses

**Figure 2:**
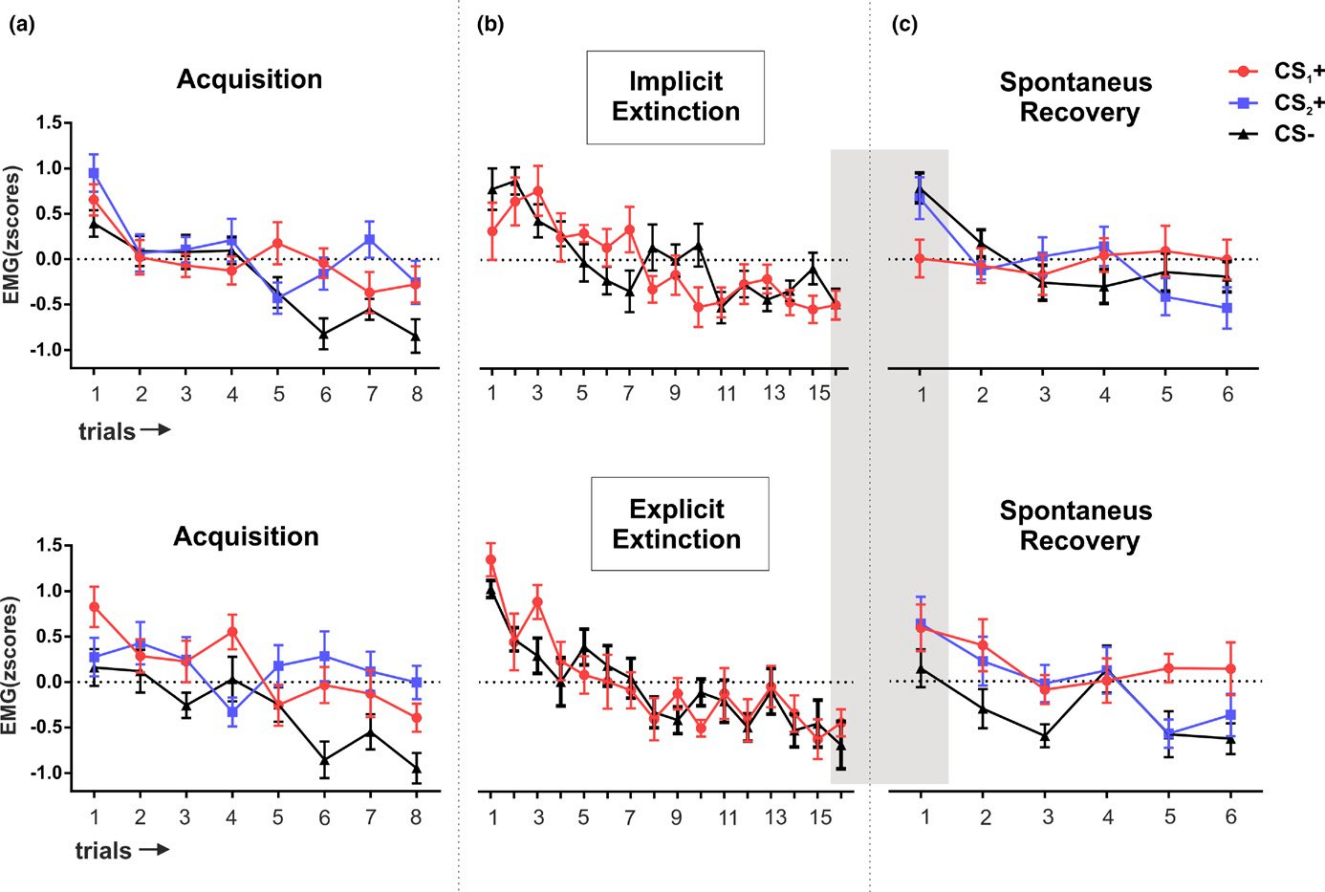
Threat‐potentiated startle responses trial by trial throughout the 3‐day experiment for both experimental groups. The top panel depicts the implicit extinction group. The lower panel depicts explicit extinction group. Mean standardized startle responses were calculated using all trials within each session for each group. Plots represent the mean response of (a) CS_1_+ CS_2_+ and CS− during acquisition on day 1, (b) CS_1_+ and CS− during extinction on day 2, and (c) CS_1_+ CS_2_+ and CS− during spontaneous recovery on day 3. EMG: electromyography. The gray shading depicts the trials that are analyzed for the recovery index. Error bars represent standard error of the mean (*SEM*)

A two‐way mixed analyses of variance (ANOVA) with group (implicit vs. explicit) as a between‐subject factor and stimuli (CS_1_+ CS_2_+ and CS−) as a within‐subject factor showed equivalent levels of SR for both groups in the last 4 trials (all *p* values >0.1 for group and group × stimuli interaction) but a main effect of stimuli (*F*_(2,66)_ = 12.23; *p* < 0.001; *η*_p_^2^ = 0.27; Figure 2a). A repeated‐measures ANOVA (RM‐ANOVA) combining both groups showed successful threat‐conditioning results: a main effect of stimuli (*F*_(2,68)_ = 12.12; *p* < 0.001; *η*_p_^2^ = 0.26) with equal responses for CS_1_+ and CS_2_+ (paired *t* test, *t*_34_ = −0.59; *p* = 0.55; *d* = 0.10) that were greater in comparison with CS− (paired *t* test CS_1_+ − CS−, *t*_34_ = 4.51; *p* < 0.001; *d* = 0.76, CS_2_+ − CS− *t*_34_ = 3.75; *p* = 0.001; *d* = 0.63).

#### 3.1.2 Electrodermal activity

**Figure 3:**
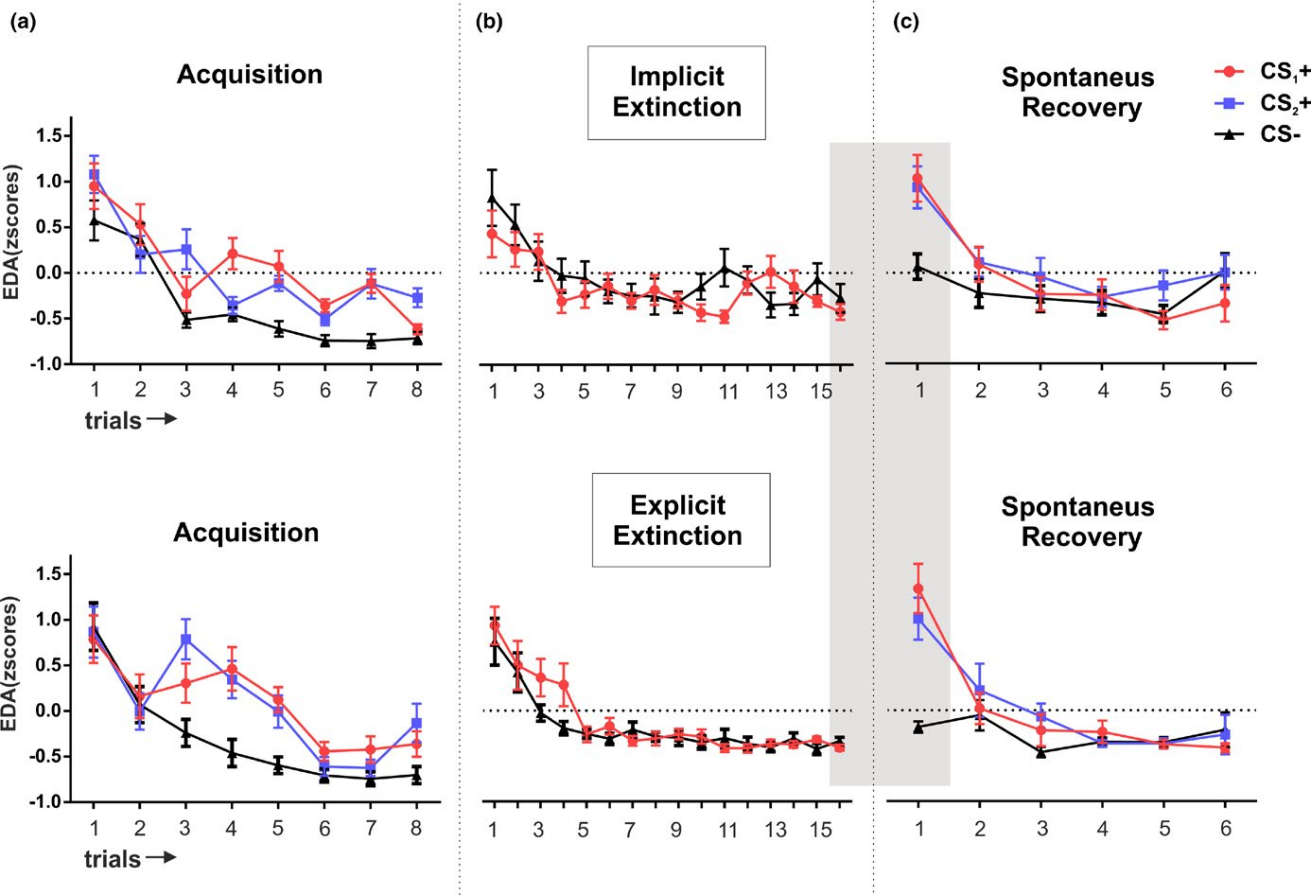
Trial‐by‐trial electrodermal activity throughout the 3‐day experiment for both experimental groups. The top panel depicts the implicit extinction group. The lower panel depicts explicit extinction group. Mean standardized electrodermal activity was quantified using all trials within each session for each group. Plots represent the mean electrodermal activity of (a) CS_1_+ CS_2_+ and CS− during acquisition on day 1, (b) CS_1_+ and CS− during extinction on day 2, and (c) CS_1_+ CS_2_+ and CS− during spontaneous recovery on day 3. EDA: electrodermal activity. The gray shading depicts the trials that are analyzed for the recovery index. Error bars represent standard error of the mean (*SEM*)

Electrodermal activity (EDA) analyses showed similar results. Responses were equivalent between groups (all *p* values >0.1 for group and group × stimulus interaction) but a main effect of stimuli was observed (*F*_(2,66)_ = 26.61; *p* < 0.001; *η*_p_^2^ = 0.44; Figure 3a). A RM‐ANOVA combining both groups showed successful threat‐conditioning results with a main effect of stimulus (*F*_(2,68)_ = 28.48; *p* < 0.001; *η*_p_^2^ = 0.45) where CS_1_+ and CS_2_+ showed equivalent responses (paired *t* test *t*_34_ = 0.29; *p* = 0.76; *d* = 0.05) but greater than the CS− (CS_1_+ − CS− *t*_34_ = 6,04; *p *< 0.001; *d* = 1.02, CS_2_+ − CS− *t*_34_ =−5.88; *p *< 0.001; *d* = 0.99).

### 3.2 Extinction session

Gradual overall decrease in responses during extinction session with no differences between groups nor between stimuli, in both measures.

We then analyzed the course of extinction learning using a two‐way mixed ANOVA with group (implicit vs. explicit) as an intersubject factor and stimulus (CS_1_+ and CS−) and time (first trials 1–2 and last trials 15–16) as intrasubject factors.

#### 3.2.1 Threat‐potentiated startle reflex

We found no differences in responses between groups nor differential responding between stimuli (all *p* values >0.5 for group, stimulus, and group × stimulus interaction). When looking at differences across time, we found a decrease in responses from beginning to end of the session (main effect of time; *F*_(1,33)_ = 55.57; *p* < 0.001; *η*_p_^2^ = 0.62) that was equivalent between groups and stimuli (all *p* values >0.1; Figure 2b).

#### 3.2.2 Electrodermal activity

Electrodermal activity analyses showed similar results, no differences between groups nor between stimuli (all *p* values >0.1 for group, stimulus, and group × stimulus interaction; Figure 3b). Again, we found a decrease in responses from beginning to end of the session (main effect of time; *F*_(1,33)_ = 57.50; *p *< 0.001; *η*_p_^2^ = 0.63) that was equivalent between groups and stimuli (all *p* values >0.1).

### 3.3 Spontaneous recovery test

**Figure 4:**
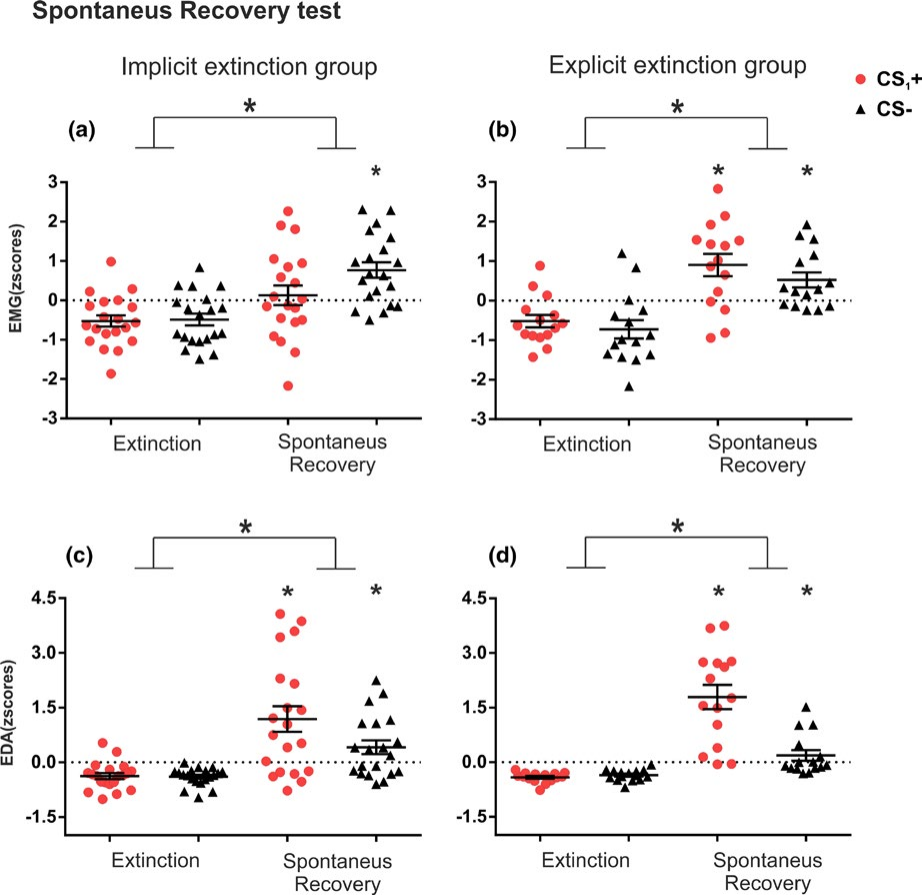
Recovery of defensive responses for both experimental groups. Mean standardized startle responses were calculated using all trials for CS_1_+ and CS− during extinction and spontaneous recovery sessions for each group in each measure. Plots represent the last trial of extinction on day 2 and the first trial of spontaneous recovery on day 3. (a) Mean of startle response for the implicit group. (b) Mean of startle response for the explicit group. (c) Mean of electrodermal activity for the implicit group. (d) Mean of electrodermal activity for the explicit group. EMG: electromyography; EDA: electrodermal activity; small*: *p* < 0.05 comparison for each stimulus between phases, big*: main effect of phase. Error bars represent standard error of the mean (*SEM*)

To test the recovery of defensive responses on day 3 we compared the last trial of the extinction session with the first trial of the spontaneous recovery test for CS_1_+ and CS− (Oyarzún et al., 2012; Schiller et al., 2010, 2013 ; Soeter & Kindt, 2011; Warren et al., 2014; Figure 4).

#### 3.3.1 Threat‐potentiated startle reflex

A two‐way mixed ANOVA with group (implicit vs. explicit) as a between‐subjects factor, and phase (extinction and recovery test) and stimulus (CS_1_+ and CS−) as within‐subject factors, revealed no main effect of group (*F*_(1,33)_ = 0.30, *p* = 0.58; *η*_p_^2^ = 0.00). A main effect of phase (*F*_(1,33)_ = 38.92; *p* < 0.001; *η*_p_^2^ = 0.54) that was equivalent between groups (phase × group_(1,33)_ = 1.06; *p* = 0.31; *η*_p_^2^ = 0.03) indicated that SR responses increased at recovery in both groups. However, we found a significant stimuli x group interaction (*F*_(1,33)_ = 10.00, *p* < 0.005; *η*_p_^2^ = 0.23; Figure 4a–b). We thus compared stimuli responses between groups. Unpaired *t* test showed similar responses for the CS‐ in both groups (*t*_(33)_ = 1.52; *p* = 1.13; *d* = 0.49) but lower responses for the CS_1_+ in the implicit than the explicit group (*t*_(33)_ = −2.19; *p* = 0.03; *d* = 0.74). Intragroup comparison of stimuli showed, in the implicit group, lower responses for the CS_1_+ in comparison with the CS− (*t*_(19)_ = −2.97; *p* = 0.008; *d* = 0.66). In contrast, similar responses for CS− and CS_1_+ were found in the explicit group (*t*_(14)_ = 1.68; *p* = 0.11; *d* = 0.43), indicating that implicit but not explicit extinction reduced SR responses to CS_1_+.

We then compared CS_1_+ responses with CS_2_+ on day 3; another homologous stimulus that was equally threat conditioned in the first session, but that was not exposed to participants on day 2 (Figure 2c). A two‐way mixed ANOVA with group (implicit vs. explicit) and stimulus (CS_1_+, CS_2_+ and CS−, standardized within day 3) as a between and within‐subject factors, respectively, revealed a significant group × stimulus interaction (*F*_(2,66)_ = 3.93; *p* = 0.02; *η*_p_^2^ = 0.11). Whereas in the explicit group, all stimuli (i.e., CS_1_+, CS−, CS_2_+) showed comparable high responses (all *p* values >0.1), differences across stimuli were found in the implicit group (implicit *F*_(2,38)_ = 3.44; *p* = 0.04; *η*_p_^2^ = 0.15, explicit *F*_(2,28)_ = 1.37; *p* = 0.26; *η*_p_^2^ = 0.09), where only CS_1_+ showed reduced response compared to the CS_2_+ (*t*_(19)_ = −2.09; *p* = 0.04, *d* = 0.46) and CS− (*t*_(19)_ = −2.77; *p* = 0.01, *d* = 0.62).

#### 3.3.2 Electrodermal activity

A two‐way mixed ANOVA with group (explicit vs. implicit) as an intersubject factor, and phase (extinction and test) and stimuli (CS_1_+ and NS) as within factors revealed a main effect of phase (*F*_(1,33)_ = 70.04; *p* < 0.001; *η*_p_^2^ = 0.69), stimuli (*F*_(1,33)_ = 15.98; *p* < 0.001; *η*_p_^2^ = 0.32), and phase × stimuli interaction (*F*_(1,33)_ = 18.02; *p* < 0.001; *η*_p_^2^ = 0.35), but no differences were found between groups (all *p* values >0.1 for group, group × stimulus and, group × stimulus × phase interaction; Figure 4c–d). We thus combined groups and compared stimuli responses between phases. As expected, responses significantly increased from the end of the extinction session to the recovery test in both stimuli (paired *t* test CS− *t*_(34)_ = −5.37; *p* < 0.001; *d* = −0.90, CS_1_+ *t*_(34)_ = −7.10; *p* < 0.001, *d* = −1.2). And, although responses between stimuli were comparable at the end of the extinction session (*t*_(34)_ = −0.40; *p* = 0.68; *d* = −0.06), responses in the recovery test were greater for CS_1_+ than for CS− (*t*_(34)_ = 3.93; *p* < 0.001; *d *= 0.66). Thus, showing that in both groups, CS_1_+ and NS, incremented EDA responses from the end of day 2 to test, but with greater recovery for CS_1_+.

We then explored whether such recovery in the CS_1_+ was similar to the response of its conditioned homologous CS_2_+ on day 3 (Figure 3c). A mixed ANOVA with group and stimuli (CS_1_+, CS_2_+, and CS−) showed no differences across groups (all *p* values >0.5 for group and group × stimulus interaction) but a main effect of stimulus (*F*_(2,66)_ = 15.21; *p* < 0.001; *η*_p_^2^ = 0.32) that was driven by equal responses for CS_1_+ and CS_2_+ on day 3 (paired *t* test *t*_(34)_ = 0.70, *p* = 0.48, *d *= 0.11) but greater than CS− (CS_1_+ − CS− *t*_(34)_ = 5.48, *p* < 0.001, *d *= 0.92, CS2 − CS− *t*_(34)_ = 5.15, *p* < 0.001,*d *= 0.87). Thus, in the EDA measure, regardless of type of extinction, conditioned stimuli CS_1_+ showed equivalent increased recovery than CS_2_+ on day 3.

### 3.4 Online Threat Expectancy Ratings (OER) on day 3

Participants’ explicit contingency learning was not modulated by either implicit or explicit extinction.

**Figure 5:**
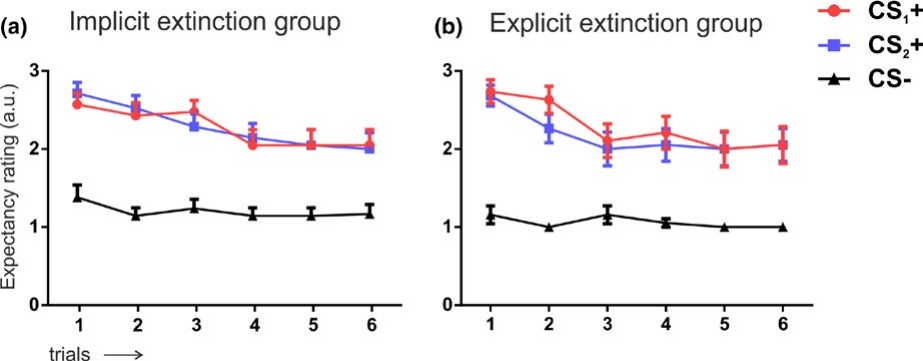
Online threat expectancy ratings during recovery test. During each picture presentation, subjects indicated whether they either expected (pressed 3), did not expect (pressed 1) or were not sure about (pressed 2) imminent shock occurrence. Error bars represent standard error of the mean (*SEM*); (a.u) arbitrary unit

We then explored on day 3 whether participants expected to be shocked after the presentation of the faces (Figure 5). A two‐way mixed ANOVA with group (implicit vs. explicit) as between‐subject factor and stimuli (CS_1_+, CS_2_+, and CS−) and time (mean of the first two trials vs. mean of the last two trials) as within‐subject factor showed no differences between groups (all *p* values >0.1 for group, group × stimuli, and group × time interaction). Thus, these results indicated that our experimental manipulation did not affect OER. However, we found a main effect of stimuli (*F*_(2,66)_ = 50.34; *p* < 0.001, *η*_p_^2^ = 0.33), time (*F*_(2,66)_ = 16.25; *p* < 0.001, *η*_p_^2^ = 0.33), and stimuli × time interaction (*F*_(2,66)_ = 5.16; *p* < 0.005, *η*_p_^2^ = 0.13). We thus explored stimuli responses across time. We found that participants’ expectancy scored for CS_1_+ and CS_2_+ stimuli decreased from beginning to the end during the recovery session (CS_1_+ *t*_(34)_ = 3.39, *p* < 0.005; *d* = 0.57, CS_2_+ *t*_(34)_ = 3.72, *p* < 0.005; *d* = 0.63). Congruent with the increased physiological responses to the CS− during early recovery, CS− showed an increase in shock expectancy at the beginning of the session (beginning to end CS− *t*_(34)_ = 2.71, *p* = .01; *d* = 0.45). Some participants reported not to be sure of expecting to be shocked when presented with the CS− (scored = 2) in the first trials. As expected, although shock expectancy was similar between CS_1_+ and CS_2_+ at both the beginning and the end of the session (all *p* values >0.5), CS− scores were significantly lower at both the beginning (CS_1_+ − CS− *t*_(34)_ = 8.90, *p* < 0.001; *d* = 1.52, CS_2_+ − CS− *t*_(34)_ = 8.44, *p* < 0.001; *d* = 1.42) and the end of the session (CS_1_+ − CS− *t*_(34)_ = 5.60, *p* < 0.001; *d* = 0.94, CS_2_+ − CS− *t*_(34)_ = 5.23, *p* < 0.001; *d* = 0.88). Thus, participants maintained the cognitive threatful representation for conditioned stimuli from the beginning to the end of the session.

## 4 PSYCHOLOGICAL INVENTORIES

### 4.1 Equivalent scores between groups

Since anxiety traits have been previously related to aspects of implicit emotional learning (Raio et al., 2012), we checked whether our participants presented equivalent scores between groups in the psychological inventories*.* No significant differences were found between groups in any of the psychological inventories (see Table 1 for descriptive statistics); participants showed similar scores in the Spanish version of the STAI‐State Inventory (unpaired *t* test; *t*_(33)_ = −0.55, *p* = 0.58, *d* = 0.19), the STAI‐Trait Inventory (*t*_(33)_ = −1.55, *p* = 0.12, *d* = 0.52), and the Spanish version of the 25‐item English Resilience with ASL and PC subscales (Group, *F*_(1,33)_ = 0.69; *p *= 0.41; *η*_p_^2^ = 0.02, group × scale interaction, *F*_(1,33)_ = 1.00; *p *= 0.32; *η*_p_^2^ = 0.03).

**Table 1:** Descriptive statistics of inventory scores

Inventory	Implicit		Explicit	
	Mean	*SD*	Mean	*SD*
STAI‐state	10.15	5.33	11.06	4.13
STAI‐trait	9.7	7.6	13.0	3.4
PC	91.22	12.56	91.46	9.04
ASL	37.11	5.77	40.26	4.11

ASL, acceptance of self and life; PC, personal competence.

These results indicate that the differences observed for the implicit and explicit groups are unlikely to be due to differences in anxiety and resilience traits between the groups.

## 5 DISCUSSION

Two groups of participants underwent a partial reinforced threat‐conditioning paradigm using three fearful faces. Two of the faces coterminated with a mild electric shock to the wrist on 75% of trials (conditioned stimuli; CS_1_+ and CS_2_+) while a third face served as the neutral stimulus (CS−).

On the second day, one group of participants underwent implicit while the other underwent explicit extinction to one of the threat‐conditioned stimuli. For the implicit condition, CS_1_+ and CS− were presented unconsciously using the continuous flash suppression (CFS) technique and no shocks were administered, while CS_2_+ was not presented. The explicit group followed the same procedure except that pictures were explicitly presented (see Materials and Methods section). On the following day, we tested spontaneous recovery, by presenting all participants explicitly with the three faces in the absence of electric shocks (see design in Figure 1). We used a combination of measures to examine defensive responses: threat‐potentiated startle reflex (SR), electrodermal activity (EDA), and online expectancy ratings (OER).

We found that exposing participants implicitly with previously threat‐conditioned stimulus reduced the recovery of defensive responses after 24 hr, measured by SR, but not by EDA or OER.

Our results highlight the divergent expression between two physiological measures (EDA and SR) where implicit extinction only modulated threat‐potentiated SR. Dissociation between both measures has long been recognized and although there is still much debate about the nature of each measure, it has been suggested that they are differently modulated by different neural systems during threat memory encoding, extinction, and retrieval (Sevenster, Beckers, & Kindt, 2014; Soeter & Kindt, 2010).

In our experiment on day 3, EDA followed a similar pattern of responses as those presented by the OER, but only at the beginning of the test session, higher responses for CS_1_+ and CS_2_+ than for CS− that gradually decreased throughout the session. Such correspondence across both measures fits well with the idea that EDA is sensitive to modulations of threat explicit expectancies (Lovibond, 2003; Sevenster et al., 2014; Soeter & Kindt, 2010). However, the fact that OER and EDA, dissociated as the session progressed; with a stronger drop in EDA to all stimuli (Figure 3) but sustained high OER (Figure 5), suggest that EDA might behave independently from contingency knowledge, as reported in other studies (Raio et al., 2012; Schultz & Helmstetter, 2010). For example, in the study of Raio et al. (2012), EDA to conditioned stimuli was observed during implicit conditioning. In addition, other studies have shown direct correlations between EDA and amygdala activity (Koizumi et al., 2016; Schiller et al., 2013), suggesting that EDA is deeply linked with the survival circuit of threat processing.

Critically, the fact that implicit extinction only modulated SR during the recovery test might suggest that, instead of tapping on a different system, SR is more sensible than EDA, to subtle modulations in the affective system, potentially induced during implicit extinction of CS_1_+. In fact, SR, as an automatic reflex, has been considered to be tightly regulated by the defensive circuit reflecting amygdala activity for negative affective valence (Hamm & Vaitl, 1996; Lang, Bradley, & Cuthbert, 1990), whereas EDA might be more sensible to cognitive modulations by the explicit expectations of upcoming relevant events (Sevenster et al., 2014; Sevenster, Beckers, & Kindt, 2012b). Critically, if this is the case, our results would suggest that implicit extinction might separately modulate the implicit trace of fearful memories.

Of note, CS− showed an increment of defensive responses in the recovery test in both groups and for both measures (when comparing the last trial of the extinction session with the first trial of the spontaneous recovery test session), suggesting a global threat generalization effect. Generalization in the physiological responses was further supported by the results in the OER where participants reported to be “not sure” of being shocked with CS− presentation in the first trials on day 3. Generalization of defensive responses in this type of paradigm has been reported previously by other studies (Kindt & Soeter, 2013; Oyarzún et al., 2012; Soeter & Kindt, 2011). In the context of our current design, it is possible that threat generalization was transferred via shared element among all stimuli (Dunsmoor & Murphy, 2015); that is, airpuffs, which were always presented at the end of each picture (to induce the blinking response) (see Materials and Methods section), were frequently followed by the electric shock (75% of times for the CSs).

An important point to consider is the fact that no differential responses between conditioned and neutral stimuli nor between groups (implicit vs. explicit) were observed throughout the course of the extinction session. One possible explanation is that the use of the stereoscope during extinction (and not during day 1 or 3) acted as a new contextual cue that limited the retrieval of threatful associations (Maren, Phan, & Liberzon, 2013) and blunted the differential responses between neutral and threat‐conditioned stimuli. The use of the stereoscope only on day 2 was aimed to increase ecological validity of the extinction task, as the acquisition of fear associations and reexposure to a fearful stimuli would be unlikely to occur throughout a stereoscope in a real context.

Our results are consistent with and build on previous studies using a very brief exposure (VBE) approach, in which pictures of spiders were presented very rapidly (i.e., 25 ms) in phobic patients, leading to a long‐lasting reduction of avoidance behavior (Siegel & Warren, 2013a, 2013b). In an attempt to look for the mechanism underlying this effect, the authors (Siegel et al., 2017) scanned patients while exposed to either masked or clear visible phobic stimuli (in two separated groups). Counterintuitively, they showed that presentations of either masked or visible phobic stimuli activated or deactivated, respectively, brain regions that support emotional regulation like ventromedial PFC. They posited that limited awareness during exposure and lack of subjective fear as well as amygdala activity reduction might facilitate fear processing and emotional regulation. In addition, in other studies, it has been shown that when the prefrontal cortex is not engaged during extinction learning (due to a lesion or due to early development stage), subjects do not present recovery of defensive responses and amygdala is more involved during extinction, leading to a permanent extinction (Kim & Richardson, 2010; Koenigs et al., 2008). These results point out the possibility that implicit extinction in our experiment might have engaged a similar mechanism that leads to attenuation of defensive responses, albeit only detected by SR measure.

Although the neural mechanism underlying CFS suppression effects is still largely unknown, a functional neuroimaging study using CFS and invisible presentations of fearful faces (Lapate et al., 2016) showed that while awareness of cues promoted PFC‐amygdala functional connectivity, invisible presentation of faces did not engage such regulatory circuit. In the case of our implicit extinction paradigm, it is possible that faces are repetitively processed by the amygdala, via a fast subcortical pathway (Méndez‐Bértolo et al., 2016) and by sensory areas representing CS while unaware and thus in the absence of activation of the defensive circuit. This, in turn, would promote emotional memory processing perhaps by the desensitization of low‐level threat‐related regions, as posited by Siegel et al. (2017). In fact, it has been reported that ex‐spider phobic patients that showed permanent extinction after 6 months presented low activity in ventro‐visual regions that were hyperresponsive to spiders before the therapy. Further, the authors revealed that reduced activity in a restricted portion of the same visual cortical region (right lateralized lingual gyrus) immediately after therapy predicted long‐term permanence of extinction learning (Hauner, Mineka, Voss, & Paller, 2012). These results suggest that tapping into sensorial and low‐level defensive networks might change the association between stimulus and defensive response, leading to permanent extinction without the need of prefrontal inhibitory control. As our experiment cannot account for any neural mechanism underlying CFS extinction, further research is still imperative to examine such an argument.

An important disadvantage and methodological limitation from CFS technique is that image suppression is often broken when presenting threatful images (Yang, Zald, & Blake, 2007) which might limit its use as a sole tool in clinical settings. In our experiment, to reduce subject attrition by image suppression failure, we implemented a task where participants had to report the color of a central dot within the Mondrian. However, the fact that participants needed to hold their answer for a couple of seconds might have comprised some cognitive demand during image presentation. It has been recently reported that working memory load during extinction can suppressed amygdala activity and subsequently reduce recovery of threat responses (Voogd, Neville, Roelofs, Fernández, & Hermans, 2018). However, the cognitive demand in this task (i.e., 27 s of a 2N‐Back task and goal‐directed eye‐movements) further exceeds the one required for our detention task. Nevertheless*,* whether this could have affected our results is unknown and more research would be needed to clear out this possibility. Despite the implementation of this task, around half of our participants needed to be ruled out in this study for having broken the suppression effect. The fact that the selection criteria eliminated so many participants might constitute a potential confound. It is possible that selected participants might share psychological features that make them different from the control group and more likely to show reduced defensive responses in the SR during spontaneous recovery. Although our selected participants rated equivalent scores in all psychological inventories, these results urge the need for further investigation and replications that could circumvent bias selection of participants by improving suppression effect during CFS extinction.

Our results deviate from those of Golkar and Öhman (2012). In their experiment, the authors extinguished two conditioned stimuli; one under masked and the other under visible conditions. In contrast to our results, in their study, the stimulus that was unconsciously extinguished presented more fear recovery than the one explicitly extinguished. However, it might well be the case that the parallel engagement of explicit and implicit learning could jeopardize the latter, as both explicit and implicit systems share encoding resources (Turk‐Browne, Yi, & Chun, 2006) and might interact in a competitive manner (Kim & Baxter, 2001). Indeed, it has been suggested that the strong cognitive component of exposure‐based therapies may actually preclude extinction learning at the implicit level (“Anxious: The Modern Mind in the Age of Anxiety by Joseph E LeDoux, book review”, 2015).

We believe that implicit extinction using CFS might promote processing of fearful memories in the subcortical threat‐related networks and facilitate emotional regulatory areas. The fact that fearful stimuli are experienced in the absence of emotional distress in patients might help to change the threatful trace and improve the course of the therapy. Although our results provide encouraging evidence supporting these ideas, our findings call out the need for further investigation to circumvent methodological limitations, precise the mechanism involved, and uncover the potential of CFS implicit extinction as a valuable complementary procedure to further advance exposure‐based psychotherapies.

## CONFLICT OF INTEREST

None declared.

## AUTHOR CONTRIBUTION

J.O. conducted the experiments and analyzed the data, J.O., R.D.B, and L.F. designed the experiments and wrote the paper, and J.O., E.C., and S.K. programmed the task.
